# *DAXX* mutations as potential genomic markers of malignant evolution in small nonfunctioning pancreatic neuroendocrine tumors

**DOI:** 10.1038/s41598-019-55156-0

**Published:** 2019-12-09

**Authors:** Mauro Cives, Stefano Partelli, Raffaele Palmirotta, Domenica Lovero, Barbara Mandriani, Davide Quaresmini, Eleonora Pelle’, Valentina Andreasi, Paola Castelli, Jonathan Strosberg, Giuseppe Zamboni, Massimo Falconi, Franco Silvestris

**Affiliations:** 1 Department of Biomedical Sciences and Human Oncology, University of Bari, Bari, Italy; 2 Pancreatic Surgery Unit, San Raffaele Scientific Institute, Milan, Italy; 3 Division of Pathology, Sacro Cuore - Don Calabria Hospital, Negrar, Italy; 4 Department of GI Oncology, Moffitt Cancer Center, Tampa, FL USA; 5 Pathology Department, University Of Verona, Verona, Italy

**Keywords:** Neuroendocrine cancer, Molecular medicine

## Abstract

Management of localized well-differentiated pancreatic neuroendocrine tumors (panNETs) is controversial and primarily dependent on tumor size. Upfront surgery is usually recommended for tumors larger than 2 cm in diameter since they frequently show metastatic potential, whereas smaller panNETs are generally characterized by an indolent clinical course, with a rate of relapse or metastasis below 15%. To explore whether increased tumor size is paralleled by genomic variations, we compared the rate and the mutational patterns of putative driver genes that are recurrently altered in these tumors by investigating differential cohorts of panNET surgical specimens smaller (n = 27) or larger than 2 cm (n = 29). We found that the cumulative number of mutations detected in panNETs >2 cm was significantly higher (*p* = 0.03) relative to smaller tumors, while mutations of *DAXX* were significantly more frequent in the cohort of larger tumors (*p* = 0.05). Moreover, mutations of *DAXX* were associated with features of malignancy including increased grade, nodal involvement and lymphovascular invasion, and independently predicted both relapse after surgery (*p* = 0.05) and reduced DFS in multivariable analysis (*p* = 0.02). Our data suggest that alterations of the *DAXX/ATRX* molecular machinery increase the malignant potential of panNETs, and that identification of mutations of *DAXX*/*ATRX* in small, nonfunctioning tumors can predict the malignant progression observed in a minority of them.

## Introduction

Pancreatic neuroendocrine tumors (panNETs) account for approximately 5% of primary pancreatic malignancies^1^. Because of the increasing use of high-quality cross-sectional imaging and endoscopy, up to 60% of panNETs are currently diagnosed when their diameter is lower than 2 cm^2,3^. These incidentally discovered, well-differentiated sporadic panNETs ≤2 cm are usually nonfunctioning and characterized by a very indolent behavior, with 5-year disease-free survival (DFS) and overall survival (OS) rates of 94% and 96% respectively^4^. Although surgical resection is considered the mainstay for the localized panNET management, its role in patients with sporadic, low-grade, asymptomatic tumors smaller than 2 cm has been recently questioned due to the substantial morbidity and mortality associated with pancreatic surgery. Consequently, a “watch and wait” policy has been formally advocated for such a selected group of patients^5,6^. On the other hand, postsurgical management studies have demonstrated that fewer than 15% of panNETs ≤2 cm exhibit malignant features such as lymph node involvement, or recurrence after resection^7–10^. Therefore, potential molecularly-defined preoperative predictors of malignancy are needed to determine whether or not surgical treatment is preferable to a clinical surveillance strategy.

Recently, whole-exome and whole-genome sequencing studies^11,12^ have provided a detailed picture of the underlying genomics of panNETs. Somatic mutations of *MEN1*, *DAXX*/*ATRX*, and mammalian target of rapamycin (mTOR) pathway genes including *PTEN*, *PIK3CA*, and *TSC2* have been reported as recurrent aberrations in panNETs’ pathogenesis and development. However, the sequential genomic abnormalities promoting the evolution from small, clinically indolent panNETs to larger, aggressive tumors are presently unclear.

Here, we investigated whether mutations of major panNET-related genes undergo a tumor biology-dependent accumulation, with a defined sequence during the growth of panNETs. Moreover, by comparing the pattern of gene mutations observed in panNETs ≤ 2 cm relative to the genomic pattern of larger sized tumors, we sought to identify molecular alterations that can predict malignant behavior.

## Results

### Demographics and tumor characteristics

Demographic variables and clinical-pathological characteristics of the 56 patients included in the study are summarized in Table 1. The median age at diagnosis was 56 (range: 36–74) and 54 (range: 35–73) in patients with panNETs ≤2 cm (*n* = 27) and >2 cm (*n* = 29) respectively. The diagnosis of panNET was most commonly incidental when tumor diameter was ≤2 cm (*p* = 0.005). The surgical treatment comprised tumor enucleation with higher frequency for panNETs ≤2 cm than for larger neoplasms (*p* = 0.03), while the number of excised lymph nodes was higher in panNETs >2 cm compared to smaller tumors (*p* = 0.03). In patients with panNETs ≤2 cm, the surgical excision was performed in response to the patient’s preference in fifteen cases, due to tumor uptake on ^18^FDG-PET in four cases, due to biliary tract dilation in three cases and due to imaging overestimation of tumor diameters in two cases. Young age at diagnosis and presence of additional suspicious intrapancreatic lesions (not further confirmed) prompted surgery in one and two patients, respectively. Features of malignancy, including advanced grade, lymph node metastasis, lymphovascular invasion and perineural invasion, were detected in a significantly higher number of panNETs >2 cm as compared with small tumors (Table 1). Of note, lymph node metastases were detected in 4 patients (14.8%; 95% CI, 5.9–32.5%) with panNET diameters of 10, 10, 14 and 15 mm respectively, in the absence of any apparent clinical, radiological or pathological features of malignancy including grade and biliary duct dilation. After a median postoperative follow-up exceeding 80 months, recurrence was diagnosed in 11/29 (37.9%; 95% CI, 22.7–56%) patients with panNETs >2 cm. The liver (*n* = 9) was the main site of metastatic relapse, with recurrences detected also in abdominal lymph nodes (*n* = 1) and bone (*n* = 1). No postoperative relapse was observed among patients with panNETs ≤2 cm.

**Table 1:** Patient demographics and tumor characteristics according to tumor size.

Characteristics	panNETs ≤2 cm (*n* = 27)	%	panNETs >2 cm (*n* = 29)	%	*p*
**Age at diagnosis (years)**					
Median	56		54		
Range	36–74		35–73		0.86
**Gender**					
Male	13	48	12	41	
Female	14	52	17	59	0.79
**Incidental diagnosis**					
Yes	22	82	13	45	
No	3	11	15	52	
Unknown	2	7	1	3	0.005
**Tumor size (cm)**					
Median	1.5		4		
Range	0.5–2		2.3–16		<0.0001
**Tumor location**					
Head	9	33	9	31	
Body	14	52	13	45	
Tail	4	15	7	24	0.68
**Surgical procedure**					
Enucleation	8	30	1	3	
Distal pancreatectomy	13	48	19	66	
Middle pancreatectomy	0	0	2	7	
Pancreaticoduodenectomy	6	22	7	24	0.03
**T stage (ENETS classification**^23^**)**					
T1	27	100	0	0	
T2	0	0	17	59	
T3	0	0	10	34	
T4	0	0	2	7	<0.0001
**N stage (ENETS classification**^23^**)**					
N0	23	85	15	52	
N1	4	15	14	48	0.007
**Lymph nodes harvested**					
Median	14		18		
Range	0–38		3–115		0.03
**Tumor grade (WHO 2017**^21^**)**					
NET G1	24	89	16	55	
NET G2	3	11	12	41	
NET G3	0	0	1	4	0.02
**Lymphovascular invasion**					
Yes	2	7	10	34	
No	25	93	19	66	0.01
**Perineural invasion**					
Yes	0	0	6	21	
No	27	100	23	79	0.01
**Recurrent disease**					
Yes	0	0	11	38	
No	27	100	18	62	0.0004
**Follow-up (months)**					
Median	84		82		
Range	57–107		20–105		0.32

### Somatic driver mutations

**Figure 1:**
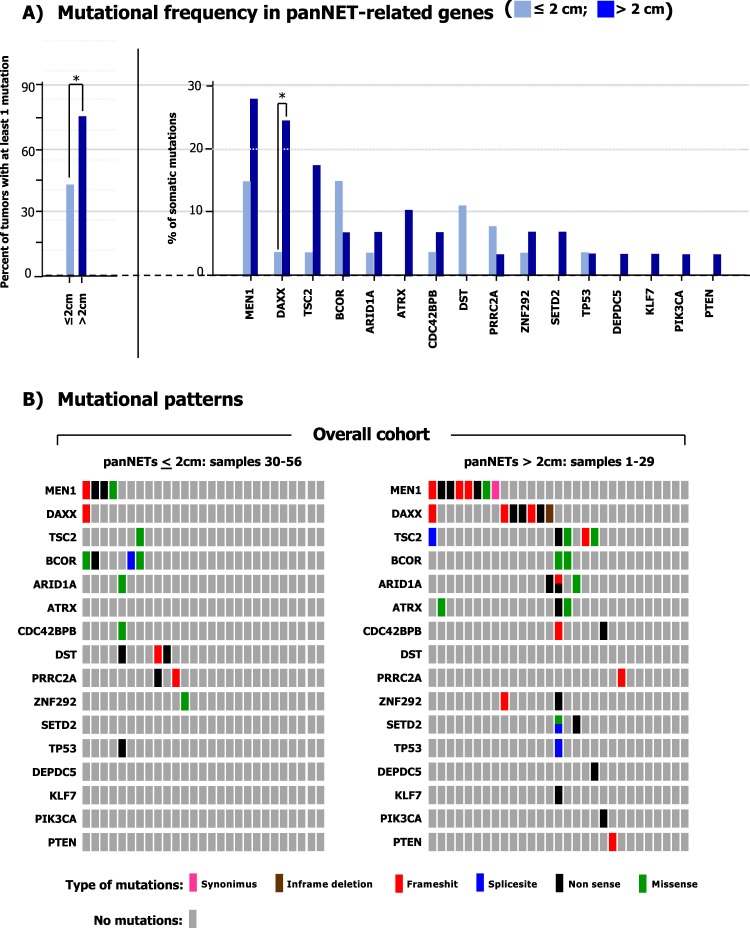
Mutational patterns in panNETs ≤2 cm and >2 cm. (**A**) The number of mutations identified in panNETs >2 cm is significantly higher than in smaller tumors. In particular, panNETs >2 cm in diameter are enriched in mutations of *DAXX*. The overall rate of mutations of *MEN1*, *DAXX* and *TSC2* was 21.4%, 14.3% and 12.5% respectively. The asterisk marks statistical significance as defined by p ≤ 0.05. (**B**) The driver plot displays the somatic mutations identified in panNETs ≤2 cm (*n* = 27) and > 2 cm (*n* = 29). Of the 19 genes sequenced, 3 genes (*MLL3*, *DIS3L2*, *URGCP*) were always wild-type, and therefore are not represented in the plot.

A total of 60 somatic mutations were detected across 16/19 genes in the 56 tumor samples subjected to targeted sequencing (Table S1). The overall number of mutations ranged between 0–4 and 0–10 in panNETs ≤2 cm and >2 cm respectively. The majority (75.9%; 95% CI, 57.9–87.8%) of panNETs >2 cm harbored at least one mutation, whereas genetic alterations were detected only in 44.4% (95% CI, 27.6–62.7%) of smaller panNETs (*p* = 0.03). As summarized in Fig. 1, *MEN1* was the most frequently mutated gene (21.4% of tumors; 95% CI, 12.7–33.8%). Inactivating mutations of *DAXX/ATRX* and mutations of genes of the mTOR pathway (*TSC2*, *DEPDC5*, *PTEN*, *PIK3CA*) were observed cumulatively in 11/56 (19.6%; 95% CI, 11.3–31.8%) and 8/56 samples respectively (14.3%; 95% CI, 7.4–25.8%). Mutations of *DAXX*/*ATRX* were always mutually exclusive, in line with prior literature^11–13^. Epigenetic controllers including *ARID1A*, *SETD2* and *BCOR* genes were mutated in 8/56 tumors (14.3%; 95% CI, 7.4–25.8%), while mutations of genes committed to cell migration such as *CDC42BPB*, *KLF7* and *DST* were cumulatively detected in 5 samples (8.9%; 95% CI, 3.9–19.3%). The genes *PRRC2A* and *ZNF292* were found mutated in 3 specimens each (5.3%; 95% CI, 1.8–14.6%). In two samples, we identified independent mutations of *ARID1A* (sample #1: two missense at 12% and 15% allelic frequency respectively; sample #23: a frameshift at 11% and a nonsense at 17% allelic frequency), thus suggesting the coexistence of putatively divergent subclones in these tumors, both having a diameter >2 cm.

### Mutational patterns of small and large panNETs

**Figure 2:**
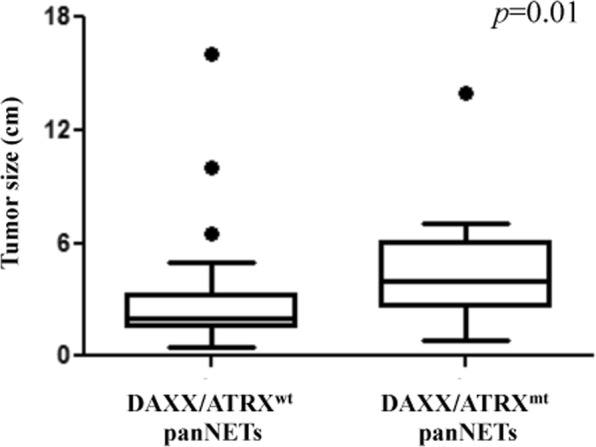
Mutations of *DAXX*/*ATRX* are associated with increased tumor size. Change in pathological tumor size according to the mutational status of *DAXX*/*ATRX*.

To explore genomic alterations which may confer clinical aggressiveness to panNETs, we compared the rate and pattern of mutations in tumors ≤2 cm and >2 cm. As shown in Fig. 1A, mutations of *DAXX* were significantly associated with increased tumor size (*p* = 0.05). When considered cumulatively, mutations of *DAXX*/*ATRX* were also significantly more frequent in panNETs >2 cm as compared with smaller tumors (*p* = 0.006). The median sizes of tumors showing an intact or a deranged *DAXX*/*ATRX* machinery were 2 cm (range: 0.5–16 cm) and 4 cm (range: 0.8–14) respectively (*p* = 0.01), as represented in Fig. 2. The single case of panNET ≤2 cm harboring a mutation of *DAXX* was graded as G2 and involved 4 out of the 21 lymph nodes excised. There was no significant difference in the mutational status of all other genes analyzed according to tumor size. Taken together, our findings suggest that mutations of *DAXX* and *ATRX* are hallmarks of tumor progression, and that they can occur rarely in panNETs ≤2 cm with pathologically-evident malignant potential.

### Gene mutations and tumor aggressiveness

**Figure 3:**
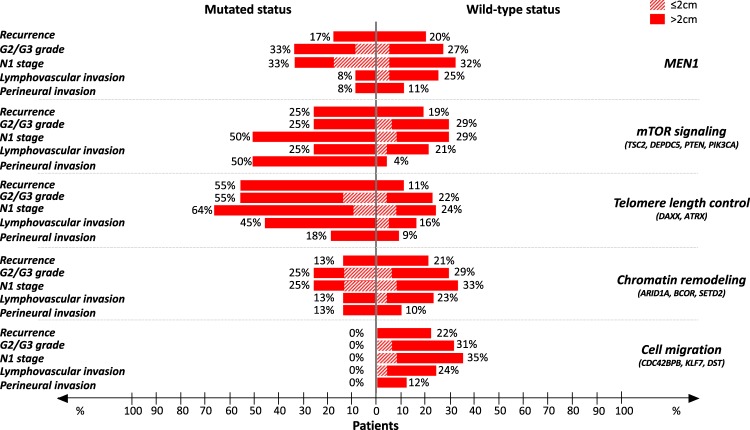
Core pathways in panNETs and associated features of malignancy. Percent of occurrence of malignancy criteria in panNET patients in relation to both size of the tumor and mutational status. Overall, alterations of the genes controlling telomere length appear to be strong predictors of tumor aggressiveness. Indeed, the rate of detection of recurrence, N1 stage, lymphovascular and perineural invasion in *DAXX/ATRX*^mt^ panNETs is considerably higher than in *DAXX/ATRX*^wt^ tumors. The presence of mutations and possibly consequent inactivation of genes committed to cell migration appears to predict reduced metastatic potential.

We evaluated the association between the rate of mutation of each gene studied and several clinical-pathological features indicative of tumor aggressiveness. In the overall cohort of panNETs (*n* = 56), mutations of *DAXX* were associated with G2/G3 grade combined (*p* = 0.03), N1 stage (*p* = 0.0009) and lymphovascular invasion (*p* = 0.008), while the mutated status of *TSC2* correlated with the presence of perineural invasion (*p* = 0.01). When considered cumulatively, mutations of *DAXX*/*ATRX* remained significantly associated with nodal involvement (*p* = 0.03) and lymphovascular invasion (*p* = 0.04), while resulting borderline associated with increased grade (*p* = 0.06). In panNETs ≤2 cm (*n* = 27), there was a trend towards correlation between *MEN1* mutations and nodal involvement (*p* = 0.09). Moreover, mutations of *DAXX* appeared to correlate with G2 grade (*p* = 0.004), although this finding should be cautiously interpreted in light of the very low number of cases analyzed (only 3 patients harbored G2 panNETs ≤2 cm). In panNETs >2 cm (*n* = 29), there was a significant association between mutations of *DAXX* and N1 stage (*p* = 0.03) and mutations of *TSC2* and perineural invasion (*p* = 0.04). The probability of detection of features of tumor malignancy according to both tumor size and mutational status of panNET-related genes is graphically represented in Fig. 3. Among the four panNETs ≤2 cm that showed a positive uptake on ^18^FDG-PET, one (sample #35) harbored a single mutation of ZNF292, while no mutations were observed in the remaining three tumors (sample #32, #34, #55).

### Gene mutations and risk of recurrence

We then assessed the impact of gene mutations on the risk of postoperative recurrence in our series. In the overall cohort of panNETs, only mutations of *DAXX* were significantly associated with tumor recurrence (*p* = 0.0003), consistent with prior literature^13,14^. After adjusting for all other variables, namely tumor size, grade, nodal involvement, lymphovascular or perineural invasion, and incidental tumor diagnosis, that were associated with disease recurrence in univariate analysis using a *p* < 0.2 threshold (Table 2), mutations of *DAXX* remained independently associated with increased risk of recurrence by multivariable analysis (odds ratio, OR = 8.3, 95% CI = 0.9–71.9; *p* = 0.05). As all recurrences occurred in patients with panNETs >2 cm, to rule out a potential confounding effect deriving from the association between tumor size and *DAXX* mutational status, we carried out a separate analysis on the sub-cohort of larger panNETs. Again, mutational status of *DAXX*, N1 stage and lymphovascular invasion were found to significantly predict relapse in panNETs >2 cm (Table S2). In multivariable analysis, after controlling for N stage, lymphovascular and perineural invasion, the association between the occurrence of *DAXX* mutations and tumor recurrence was borderline retained (OR = 12.6, 95% CI = 0.7–223.1; *p* = 0.08).

**Table 2:** Predictors of panNET recurrence: univariate and multivariable analysis.

	Recurrent disease (*n* = 11)	No recurrent disease (n = 45)	*p* (univariate)	*p* (multivariable)
DAXX			0.0003	0.05
Wild-type	5	43		(OR = 8.3; 95% CI, 0.9–71.9)
Mutated	6	2		
N stage			<0.0001	0.005
N0	1	37		(OR = 26.2; 95% CI, 2.7–255.5)
N1	10	8		
Lymphovascular invasion			0.0008	—
Present	7	5		
Absent	4	40		
Perineural invasion			0.01	—
Present	4	2		
Absent	7	43		
Grade			0.06	—
G1	5	35		
G2/G3	6	10		
Tumor location			0.64	—
Head	4	14		
Body	4	23		
Tail	3	8		
Tumor size			0.003	—
≤2 cm	0	27		
>2 cm	11	18		
Incidental diagnosis			0.03	—
Yes	4	31		
No	7	11		

### Gene mutations and survival outcomes

**Figure 4:**
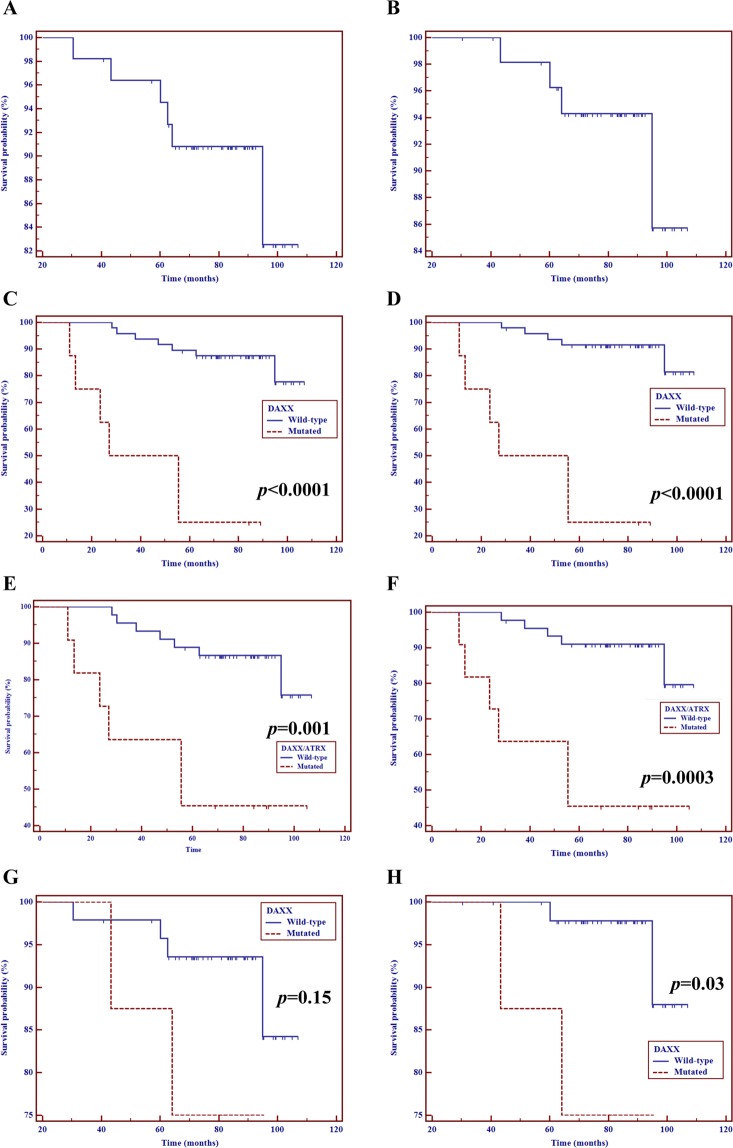
Disruption of the *DAXX/ATRX* machinery predicts poor prognosis in patients with panNETs. Kaplan-Meier estimates of DFS (**A**) and TTP (**B**) in the overall population. DFS (**C**), TTP (**D**), OS (**G**) and CSS (**H**) according to *DAXX* mutational status. DFS (**E**) and TTP (**F**) according to *DAXX/ATRX* mutational status. As depicted, somatic mutations of *DAXX* correlate with a negative impact on both DFS, TTP and CSS in patients with panNETs.

Both median DFS and TTP were not reached after a median follow-up of 83.7 months. The 5- and 10-year DFS rates were 80.4% (± 5.3%) and 69.8% (± 9.6%), while the 5- and 10-year TTP rates were 82% (± 5.2%) and 72.9% (± 9.7%). As illustrated in Fig. 4, patients who harbored a *DAXX*^wt^ panNET had a superior 5-year TTP rate as compared with those who had a *DAXX*^mt^ tumor (91.5% ± 4% versus 25% ± 15.3% respectively; *p* < 0.0001). Similarly, mutations of *DAXX* predicted significantly shorter DFS (5-year DFS: 89.6% ± 4.4% in wild-type neoplasms versus 25% ± 15.3% in mutated tumors; *p* < 0.0001). When considered cumulatively, mutations of *DAXX* and *ATRX* were similarly predictive of TTP (*p* = 0.0003) and DFS (*p* = 0.001). In multivariable analysis, mutations of *DAXX* remained independently associated with poorer DFS and TTP after controlling for tumor size, grade, nodal involvement, lymphovascular or perineural invasion (*p* = 0.02 and *p* = 0.01, respectively). The mutational status of all other genes analyzed was not associated with significant changes in either DFS or TTP. In the sub-cohort of panNETs >2 cm, mutations of DAXX significantly predicted reduced DFS (*p* = 0.04) and TTP (*p* = 0.03) (data not shown).

We finally evaluated the prognostic impact of gene mutations on survival of panNET patients. Six deaths occurred among the 56 patients enrolled. Of the 4 deaths recorded among relapsing patients, all were directly attributable to progressive metastatic disease. The 10-year OS and CSS rates were 82.5% (± 8.6%) and 85.7% (± 8.7%), respectively. Tumor size >2 cm predicted reduced OS (*p* = 0.01) and CSS (*p* = 0.05) (Fig. S1). As depicted in Fig. 4G,H, mutations of *DAXX* were associated with worse OS (*p* = 0.15) and CSS (*p* = 0.03), although this effect was not retained in multivariable analysis. All the other genes analyzed did not show any prognostic potential, and the lack of impact on survival outcomes persisted even after grouping genes according to their function (mTOR pathway, epigenetic remodeling, cell migration).

## Discussion

The clinical behavior of panNETs is critically influenced by tumor size. While tumors >2 cm in diameter frequently show malignant behavior, smaller neoplasms tend to have a more indolent clinical course and no uniform consensus for radical resection is shared among clinicians and surgeons. So far, the molecular properties of such a difference have been poorly investigated, and it remains unclear whether panNETs smaller or larger than 2 cm constitute genomically distinct tumor entities.

In this work, we compared the genomics of small nonfunctioning panNETs with that of larger nonfunctioning tumors to define the sequence of mutational events driving the progression from slow-growing, clinically benign neoplasms to aggressive malignancies capable of metastasizing. By analyzing the overall rate of mutations in our cohort, we found that the number of panNETs >2 cm showing at least one mutation is approximately double than that of panNETs ≤2 cm. This is consistent with the idea that a progressive accumulation of mutations occurs during the natural history of panNETs or, alternatively, that small and large panNETs follow distinct biologic pathways in their development. In agreement with our finding, an independent analysis of the cohort of 85 nonfunctioning panNETs subjected to whole-genome sequencing by Scarpa *et al*.^12^ reveals that the whole-genome mutational rate in large tumors (*n* = 71) is significantly higher as compared with panNETs ≤2 cm (*n* = 14), with a median number of mutations/genome of 2,297 and 1,023 respectively (*p* = 0.009) as well as a median number of non-silent mutations/genome of 21 and 11.5 respectively (*p* = 0.03; Fig. S2).

The observation that a correlation exists between mutation frequency and tumor size might have relevant implications. First, since nonfunctioning panNETs ≤2 cm and panNETs >2 cm have a median mutational load (when calculated on the whole genome) of 0.32/Mb (range 0.04–3.04) and 0.71/Mb (range 0.15–4.56) respectively^12^, a direct correlation between genomic instability and clinical aggressiveness may be envisaged. Second, given the linear relationship (Fig. S2B) between mutations and tumor dimension, we may speculate that differently-sized nonfunctioning panNETs constitute a spectrum of biologically different diseases in which the tumor enlargement is a proxy for a possible continuum of biologic aggressiveness. Consistent with this idea, in a recent retrospective, multi-institutional series of 210 patients with surgically resected small nonfunctioning panNETs, postoperative recurrence was reported only in tumors larger than 1 cm in diameter, but not in smaller neoplasms (*n* = 59)^4^, thus suggesting that even within the group of small panNETs the risk of malignant behavior is apparently related to the increase of the tumor size.

Two distinct pathways, namely chromosomal instability (CIN) or microsatellite instability (MSI), may cause the progressive accumulation of mutations within a tumor. Accumulating evidence suggests that the deficiency of the mismatch repair machinery is uncommon in well-differentiated panNETs^15^, while CIN has been identified in a substantial fraction of these malignancies^13,16^. Previous research in panNETs^13^ demonstrated that CIN is the result of the activation of the alternative lengthening of telomeres (ALT) pathway, a telomerase-independent mechanism of telomere maintenance, and multiple studies showed a robust correlation between tumor size, ALT activation, and loss of DAXX/ATRX, mainly as consequence of gene mutations^13,14,17^. In our study, we provide evidence that mutations of *DAXX*/*ATRX* occur almost exclusively in panNETs >2 cm and that mutations of *DAXX* particularly correlate with well-established features of malignancy including increased grade, lymphovascular invasion and nodal involvement. Of note, no difference was observed in our cohort in terms of rate of mutations of the other genes investigated, thus suggesting that these alterations may occur early and only marginally influence the panNET malignant behavior. Consistently, similar results can be also achieved by analyzing the cohort of nonfunctioning panNETs investigated by Scarpa *et al*.^12^, where an association between mutations of *DAXX* and both increased tumor size (*p* = 0.1; Fig. S3), increased grade (*p* = 0.07) and lymphovascular invasion (*p* = 0.03) was observed, in the absence of any trend to correlation between other genes and tumor size.

In keeping with the available literature, our findings allow us to update as follows the model of panNET progression originally proposed by Marinoni *et al*.^13^: (i) mutations of *MEN1*, chromatin remodelers (*ARID1A*, *SETD2*, *BCOR*) and mTOR pathway genes occur early in the development of panNETs, and may thus represent driver events in the initiation of these neoplasms; (ii) secondary mutations of *DAXX*/*ATRX* trigger ALT and CIN, thereby leading to the progressive accumulation of mutations and chromosomal aberrations, with consequent acquisition of malignant behavior and clinical aggressiveness; (iii) the *DAXX*/*ATRX* mutated status and the ALT-positive phenotype tends to be retained by tumor cells within metastases^17,18^, probably conferring a survival advantage over non-mutated subclones. Although intriguing, this model shows several limitations, and some of them are highlighted by our study. First, not all panNETs harbor identifiable mutations of the genes putatively involved in primary development of these malignancies, thus implying that alternative mechanisms may also drive tumor formation. For example, in our cohort, 15/27 (55%) panNETs ≤2 cm and 7/29 (24%) tumors >2 cm did not show any mutations in the panel of genes investigated. Second, although mutations of *DAXX*/*ATRX* critically enhance tumor malignancy, tumor relapse and metastasis formation may also occur in the setting of tumors with an intact *DAXX*/*ATRX* machinery, as observed in our study as well as in others^13,17–19^. Additional biological mechanisms, or alternative genetic alterations, may probably contribute to the progression of such a specific subset of panNETs that do not follows the “canonical” model of multistep evolution.

We also revealed that mutations of *DAXX* may independently predict postoperative recurrence and reduced DFS. This is not surprising, as multiple studies have already shown the negative impact of *DAXX/ATRX* loss on patient prognosis^13,14,17,19,20^. Notably, all the remaining genes investigated in the present study, even when clustered in functional groups, showed no influence on survival outcomes. Although our series was not powered enough to detect small differences in survival outcomes due to low numbers, stratification by genes other than *DAXX*, led to superimposed Kaplan-Meier curves in most cases. This further emphasizes the critical importance of the mutational status of *DAXX*/*ATRX* in modulating the clinical behavior of panNETs.

Although our ability to detect genomic predictors of malignancy in our cohort of panNETs ≤2 cm was apparently weakened by the absence of recurrences and detection of nodal involvement in only three patients, it appears that small tumors with deranged *DAXX/ATRX* molecular machinery are likely to undergo aggressive behavior. Accordingly, the single small panNET mutated for *DAXX* in our series presented with lymph node metastasis.

Potential limitations of our study include the number of samples as well as the exclusive analysis of patients who had undergone surgery. Indeed, the exclusion of patients undergoing surveillance may lead to overestimation of the malignant potential of tumors ≤2 cm due to selection bias. However, it is important to note that the majority (60%) of patients with panNETs ≤2 cm underwent resection because of patient preference, and not medical indication, thereby limiting the risk of preselecting patients with enhanced tumor aggressiveness. More importantly, our analysis focused exclusively on the targeted sequencing of genes frequently mutated in panNETs, and therefore we cannot rule out that other genetic derangements, such as cytogenetic abnormalities, copy number changes, or alterations of genes not included in our experimental model, are involved in the evolution from small, benign panNETs to large, aggressive ones.

In conclusion, our study demonstrates that a progressive accumulation of mutations occurs during the growth of panNETs. In particular, the acquisition of *DAXX* mutations usually marks the progression from small tumors lacking metastatic potential to larger neoplasms with malignant behavior. Although further research is needed to identify molecular predictors of malignancy, we propose that the determination of the *DAXX*/*ATRX* status in biopsy specimens from patients with small nonfunctioning sporadic panNETs may help tailor treatments for this difficult-to-manage group of patients.

## Patients and Methods

### Patient selection

We searched a retrospective database of patients with sporadic, well-differentiated, nonfunctioning, unifocal panNETs who had undergone R0 surgery at the Ospedale Sacro Cuore-Don Calabria, Verona (Italy) between January 2009 and December 2012. Patients who had undergone systemic anticancer therapy before resection, patients who were lost to follow-up immediately after the surgery, as well as those with metastatic disease at diagnosis were excluded from the study. Furthermore, patients with genetic syndromes associated with panNETs (i.e., multiple endocrine neoplasia type 1 syndrome, von-Hippel Lindau syndrome, neurofibromatosis type 1 syndrome, tuberous sclerosis complex syndrome, etc.) were also excluded. Demographic, clinical and pathological information were obtained by reviewing medical records. Approval for data collection and analysis was obtained from the Ethics Committee of the University of Bari (n. 5129), the IRB of the Moffitt Cancer Center (MCC 18676) and the Ethics Committee of Verona (n. 61657). Patients signed an informed consent to the use of their biological samples according to the Ethics Committee determination. All the data were handled according to the local Institutional Review Boards of each participating center. The study was performed in accordance with the principles of the Declaration of Helsinki.

### Pathological analysis and DNA extraction

Tumor specimen slides were reviewed for each case by the study pathologists (GZ, PC) in order to (i) confirm the diagnosis of NET, (ii) grade tumors according to the World Health Organization (WHO) 2017 criteria^21^, and (iii) select the formalin-fixed paraffin-embedded (FFPE) sections with a tumor cell density >80%. Thus, DNA was extracted from FFPE sections of 10 μm in thickness using the QIAamp DNA FFPE Tissue Kit (Qiagen, Germantown, MD) and quantified by Qubit DNA HS Assay (Thermo Fisher Scientific, Waltham, MA). The purity of the DNA was measured by NanoDrop ND-2000 (Thermo Fisher Scientific).

### Targeted next-generation sequencing

A group of genes known to be recurrently mutated in panNETs, namely *MEN1*, *DAXX*, *ATRX*, *PTEN*, *PIK3CA*, *TSC2*, *TP53*, *CDC42BPB*, *DST*, *DEPDC5*, *KLF7*, *BCOR*, *PRRC2A*, *URGCP*, *ARID1A*, *ZNF292*, *DIS3L2*, *MLL3*, and *SETD2*^11,12^ were subjected to targeted next-generation sequencing using an Ion AmpliSeq Custom Panel (Ion AmpliSeq Designer version 6.1.2; Thermo Fisher Scientific), as described^22^. Briefly, 10 ng of DNA were amplified and barcoded using Ion AmpliSeq Library kit 2.0 and Ion Xpress barcode adapters (Thermo Fisher Scientific). Template preparation was performed with the Ion OneTouch 2 System and Ion OneTouch ES, while sequencing was run on an Ion Torrent Personal Genome Machine (PGM; Thermo Fisher Scientific) using Ion Hi-Q View Sequencing kit (Thermo Fisher Scientific) and 318v2 chips. Postsequencing data analyses including alignment to the hg19 human reference genome as well as variant calling, were performed using the Torrent Suite Software v5.0.4 and the Torrent Variant Caller v5.10.3.0. Moreover, alignments and putative mutations were visually verified using the Integrative Genomics Viewer v2.3 (Broad Institute, Cambridge, MA). Each variant with an allele frequency ≥10% and a sequencing depth of at least 30X was investigated in its potential pathogenic role using both prediction algorithms including SIFT, Provean and Polyphen as well as publicly available databases such as COSMIC, Ensembl, HGMD, LOVD, Alfred, ExAC and dbSNP. The average base coverage depth for most samples was more than 1,000 reads.

### Statistical analysis

Descriptive statistics were used for patient demographics. The distribution of covariates including the rate and pattern of mutations was compared between panNETs ≤2 cm and panNETs > 2 cm using Fisher’s test for categorical variables and Mann-Whitney test for continuous data. Disease-free survival (DFS) was calculated from date of surgery until evidence of recurrence or death. Time to progression (TTP) was measured from date of surgery until evidence of recurrence. Overall survival (OS) was calculated from the date of diagnosis until death from any cause or until the last known follow-up. Cancer-specific survival (CSS) was also considered from the diagnosis until the cancer-related death or to last known follow-up. All time-to-event functions were estimated by the Kaplan-Meyer method and compared by the log-rank test. Multivariable analysis was performed using Cox proportional hazards regression. The assumption of proportionality was verified by log-log plot. Only variables with a *p* < 0.2 on univariate analysis were included in the Cox model. Exact 95% confidence intervals (CIs) were calculated for each proportion of interest. All tests were two-sided and statistical significance was declared at a *p* ≤ 0.05. Statistical analysis was performed using MedCalc 12.7 (MedCalc Software bvba, Ostend, Belgium).

## Supplementary information

Supplementary information
