# Supplementary material for: DAXX mutations as potential genomic markers of malignant evolution in small nonfunctioning pancreatic neuroendocrine tumors
Source: Sci Rep. 2019 Dec 9;9:18614. doi: 10.1038/s41598-019-55156-0 (PMC6901561; doi:10.1038/s41598-019-55156-0)

***DAXX* mutations as potential genomic**

**markers of malignant evolution in small nonfunctioning pancreatic neuroendocrine tumors**

**Cives Mauro, Partelli Stefano, Palmirotta Raffaele, Lovero Domenica, Mandriani Barbara, Quaresmini Davide, Pelle’ Eleonora, Andreasi Valentina, Castelli Paola, Strosberg Jonathan, Zamboni Giuseppe, Falconi Massimo, Silvestris Franco**

**Table S1 – Targeted sequencing of panNETs >2 cm (samples 1-29) and ≤2 cm (samples 30-56): summary of mutations.**

| Sample | Gene | Transcript Accession | Nucleotide  (genomic) | Nucleotide  (cDNA) | AA (protein) | Mutation  Type | COSMIC |
| --- | --- | --- | --- | --- | --- | --- | --- |
| #1 | *ARID1A* | [CCDS285.1](https://genome.ucsc.edu/cgi-bin/hgc?hgsid=689567145_SSvBKr9MmFh8FBm3t5I6McOaiz6D&g=ccdsGene&i=CCDS285.1&c=chr1&o=27022521&l=27022521&r=27108601&db=hg19) | g.chr1:27106858G>A | c.6469G>A | p.Asp2157Asn | Missense | COSM299663 |
|  | *ARID1A* | [CCDS285.1](https://genome.ucsc.edu/cgi-bin/hgc?hgsid=689567145_SSvBKr9MmFh8FBm3t5I6McOaiz6D&g=ccdsGene&i=CCDS285.1&c=chr1&o=27022521&l=27022521&r=27108601&db=hg19) | g.chr1:27101620G>A | c.4902G>A | p.Met1634Ile | Missense | COSM5443641 |
|  | *ATRX* | CCDS14434.1 | g.chrX:76875998G>A | c.5137C>T | p.Pro1713Ser | Missense |  |
|  | *BCOR* | CCDS48093.1 | g.chrX:39932804C>T | c.1795G>A | p.Gly599Ser | Missense | COSM6960684 |
|  | *TSC2* | CCDS10458.1 | g.chr16:2130166G>A | c.3398G>A | p.Gly1133Glu | Missense |  |
| #2 | *MEN1* | CCDS31600.1 | g.chr11:64577147G>A | c.435C>T | p.Ser145Ser | Synonymous | COSM5020099 |
| #3 | *SETD2* | CCDS2749.2 | g.chr3:47125359C>A | c.5911G>T | p.Glu1971Ter | Nonsense |  |
| #4 | *DEPDC5* | CCDS46692.1 | g.chr22:32275522C>T | c.3817C>T | p.Gln1273Ter | Nonsense |  |
|  | *TSC2* | CCDS10458.1 | g.chr16:2130166G>A | c.3398G>A | p.Gly1133Glu | Missense |  |
| #5 | *DAXX* | CCDS4776.1 | g.chr6:33289038G>T | c.550G>T | p.Glu184Ter | Nonsense |  |
| #6 | *DAXX* | CCDS4776.1 | g.chr6:33289038G>T | c.550G>T | p.Glu184Ter | Nonsense |  |
| #9 | *PTEN* | CCDS31238.1 | g.chr10:89624241delAA | c.17_18delAA | p.Lys6fs | Frameshift | COSM4929 |
| #10 | *DAXX* | CCDS4776.1 | g.chr6:33287329delC | c.1803delC | p.Arg602fs | Frameshift |  |
|  | *MEN1* | CCDS31600.1 | g.chr11:64577424delG | c.157delG | p.Val53fs | Frameshift | COSM85841 |
|  | *TSC2* | CCDS10458.1 | g.chr16:2110815G>A | c.1119+1G>A | c.1119+1G>A | SpliceSite | COSM6864552 |
| #11 | *MEN1* | CCDS31600.1 | g.chr11:64573133G>T | c.1174G>T | p.Glu392Ter | Nonsense | COSM22571 |
| #12 | *MEN1* | CCDS31600.1 | g.chr11:64577392C>T | c.190C>T | p.Gln64Ter | Nonsense | COSM6921970 |
|  | *ATRX* | CCDS14434.1 | g.chrX:76875970T>C | c.5165A>G | p.His1722Arg | Missense |  |
| #13 | *DAXX* | CCDS4776.1 | g.chr6:33286832_33286833insT | c.2140_2141insT | p.Ser714fs | Frameshift |  |
| #14 | *PRRC2A* | CCDS4708.1 | g.chr6:31604040_31604041insG | c.5682_5683insG | p.Leu1895fs | Frameshift |  |
| #15 | *MEN1* | CCDS31600.1 | g.chr11:64574529delAGATCTAGAGGAGC | c.867_880delAGATCTAGAGGAGC | p.Asp290fs | Frameshift |  |
| #16 | *MEN1* | CCDS31600.1 | g.chr11:64575041delG | c.780delG | p.Glu260fs | Frameshift |  |
| #17 | *DAXX* | CCDS4776.1 | g.chr6:33288636C>T | c.952C>T | p.Arg318Ter | Nonsense | COSM3349810 |
| #19 | *DAXX* | CCDS4776.1 | g.chr6:33287930delTGA | c.1356_1358delTGA | p.Asp452del | Non-frameshift Deletion |  |
| #23 | *ARID1A* | CCDS285.1 | g.chr1:27057976C>T | c.1684C>T | p.Gln562Ter | Nonsense | COSM6927396 |
|  | *ARID1A* | [CCDS285.1](https://genome.ucsc.edu/cgi-bin/hgc?hgsid=689567145_SSvBKr9MmFh8FBm3t5I6McOaiz6D&g=ccdsGene&i=CCDS285.1&c=chr1&o=27022521&l=27022521&r=27108601&db=hg19) | g.chr1:27087510insT | c.2086_2087insT | p.Ser696fs | Frameshift | COSM4170635 |
|  | *ATRX* | CCDS14434.1 | g.chrX:76764035G>A | c.7273C>T | p.Gln2425Ter | Nonsense |  |
|  | *BCOR* | CCDS48093.1 | g.chrX:39933582C>T | c.1016C>T | p.Pro339Leu | Missense |  |
|  | *CDC42BPB* | CCDS9978.1 | g.chr14:103406236_103406237insCAGA | c.4638_4639insCAGA | p.Met1547fs | Frameshift |  |
|  | *TSC2* | CCDS10458.1 | g.chr16:2129625C>T | c.3352C>T | p.Gln1118Ter | Nonsense | COSM6966671 |
|  | *KLF7* | CCDS59438.1 | g.chr2:207988567C>T | c.580C>T | p.Gln194Ter | Nonsense |  |
|  | *SETD2* | CCDS2749.2 | g.chr3:47161671C>T | c.4454+1G>A | c.4454+1G>A | SpliceSite | COSM480165 |
|  | *TP53* | CCDS11118.1 | g.chr17:7577609C>G | c.673-1G>C | c.673-1G>C | SpliceSite |  |
|  | *ZNF292* | [CCDS47457.1](https://genome.ucsc.edu/cgi-bin/hgc?hgsid=689567145_SSvBKr9MmFh8FBm3t5I6McOaiz6D&g=ccdsGene&i=CCDS47457.1&c=chr6&o=87865268&l=87865268&r=87973406&db=hg19) | g.chr6:87969405C>T | c.6058C>T | p.Gln2020Ter | Nonsense |  |
| #24 | *CDC42BPB* | CCDS9978.1 | g.chr14:103412005C>T | c.3805C>T | p.Arg1269Ter | Nonsense |  |
|  | *PIK3CA* | [CCDS43171.1](https://genome.ucsc.edu/cgi-bin/hgc?hgsid=689567145_SSvBKr9MmFh8FBm3t5I6McOaiz6D&g=ccdsGene&i=CCDS43171.1&c=chr3&o=178866310&l=178866310&r=178952497&db=hg19) | g.chr3:178943827C>T | c.2494C>T | p.Arg832Ter | Nonsense | COSM4481256 |
| #26 | *TSC2* | CCDS10458.1 | g.chr16:2136813delA | c.4931delA | p.Asp1644fs | Frameshift |  |
| #27 | *MEN1* | CCDS31600.1 | g.chr11:64572558T>G | c.1313A>C | p.His438Pro | Missense | COSM6976420 |
| #28 | *MEN1* | CCDS31600.1 | g.chr11:64573130G>T | c.1177G>T | p.Glu393Ter | Nonsense | COSM22604 |
| #29 | *DAXX* | CCDS4776.1 | g.chr6:33287598delC | c.1534delC | p.Leu512fs | Frameshift |  |
|  | *ZNF292* | [CCDS47457.1](https://genome.ucsc.edu/cgi-bin/hgc?hgsid=689567145_SSvBKr9MmFh8FBm3t5I6McOaiz6D&g=ccdsGene&i=CCDS47457.1&c=chr6&o=87865268&l=87865268&r=87973406&db=hg19) | g.chr6:87925664del T | c.215delT | p.Leu72fs | Frameshift |  |
| #35 | *ZNF292* | [CCDS47457.1](https://genome.ucsc.edu/cgi-bin/hgc?hgsid=689567145_SSvBKr9MmFh8FBm3t5I6McOaiz6D&g=ccdsGene&i=CCDS47457.1&c=chr6&o=87865268&l=87865268&r=87973406&db=hg19) | g.chr6:87967002G>T | c.3655G>T | p.Asp1219Tyr | Missense |  |
| #36 | *PRRC2A* | CCDS4708.1 | g.chr6:31592072C>T | c.325C>T | p.Gln109Ter | Nonsense |  |
|  | *DST* | CCDS75474.1 | g.chr6:56397172G>A | c.10721G>A | p.Trp3574Ter | Nonsense |  |
| #38 | *PRRC2A* | CCDS4708.1 | g.chr6:31604589_31604590insT | c.6015_6016insT | p.Pro2006fs | Frameshift |  |
| #39 | *DST* | CCDS75474.1 | g.chr6:56418347_56418348insA | c.8885_8886insA | p.Asn2962fs | Frameshift |  |
| #43 | *BCOR* | CCDS48093.1 | g.chrX:39914620G>A | c.4741+1G>A | c.4741+1G>A | SpliceSite |  |
| #45 | *MEN1* | CCDS31600.1 | g.chr11:64575070delT | c.751delT | p.Ser251fs | Frameshift | COSM255133 |
|  | *DAXX* | CCDS4776.1 | g.chr6:33289047_33289048insC | c.540_541insC | p.Thr181fs | Frameshift |  |
|  | *BCOR* | CCDS48093.1 | g.chrX:39923149C>T | c.3559C>T | p.His1187Tyr | Missense | COSM211414 |
| #47 | *DST* | CCDS75474.1 | g.chr6:56420495G>A | c.8427G>A | p.Trp2809Ter | Nonsense |  |
|  | *TP53* | CCDS11118.1 | g.chr17:7573967C>T | c.1060C>T | p.Gln354Ter | Nonsense | COSM6081949 |
|  | *CDC42BPB* | CCDS9978.1 | g.chr14:103410270G>A | c.4366C>T | p.Arg1456Cys | Missense | COSM5442819 |
|  | *ARID1A* | [CCDS285.1](https://genome.ucsc.edu/cgi-bin/hgc?hgsid=689567145_SSvBKr9MmFh8FBm3t5I6McOaiz6D&g=ccdsGene&i=CCDS285.1&c=chr1&o=27022521&l=27022521&r=27108601&db=hg19) | g.chr1:27107078T>C | c.6689T>C | p.Met2230Thr | Missense | COSM6919186 |
| #50 | *MEN1* | CCDS31600.1 | g. chr11:64573708C>T | c.1045C>T | p.Gln349Ter | Nonsense | COSM23009 |
| #51 | *BCOR* | CCDS48093.1 | g.chrX:39933336_39933337insCGA | c.1260_1262delTGGinsCGA | p.Gly421Asp | Missense | COSM6441948 |
| #52 | *BCOR* | CCDS48093.1 | g.chrX:39921487C>T | c.4333C>T | p.Gln1445Ter | Nonsense |  |
|  | *MEN1* | CCDS31600.1 | g.chr11:64577497C>T | c.85C>T | p.Arg29Ter | Nonsense | COSM6921464 |
| #53 | *MEN1* | CCDS31600.1 | g.chr11:64572612G>C | c.1259G>C | p.Arg420Pro | Missense |  |
| #56 | *TSC2* | CCDS10458.1 | g.chr16:2129658C>T | c.3385C>T | p.Arg1129Cys | Missense | COSM6940589 |

**Table S2** – Predictors of recurrence in radically resected panNETs >2 cm.

|  | Recurrent disease  (*n*=11) | No recurrent disease  (n=18) | *p*  (univariate) | *p*  (multivariable) |
| --- | --- | --- | --- | --- |
| DAXX  Wild-type  Mutated | 5  6 | 17  1 | 0.005 | 0.08  (OR=12.6; 95% CI, 0.7-223.1) |
| N stage  N0  N1 | 1  10 | 14  4 | 0.0005 | 0.01  (OR=25.6; 95% CI, 2-321.5) |
| Lymphovascular invasion  Present  Absent | 7  4 | 3  15 | 0.02 | - |
| Perineural invasion  Present  Absent | 4  7 | 2  16 | 0.16 | - |
| Grade  G1  G2/G3 | 5  6 | 11  7 | 0.41 | - |
| Tumor location  Head  Body  Tail | 4  4  3 | 5  9  4 | 0.77 | - |
| Incidental diagnosis  Yes  No | 4  7 | 9  8 | 0.39 | - |

**Supplementary figures legend**

**Figure S1 – Increased tumor size predicts poor prognosis in patients with panNETs.** Kaplan-Meier estimates of OS (A) and CSS (B) according to tumor size.

**Figure S2 – Mutational burden of panNETs ≤2 cm and >2 cm.** In the cohort of 85 panNETs assessed by Scarpa *et al.* [12], panNETs ≤2 cm appear to have a significantly lower mutational burden as compared with larger tumors. (A) Relationship between the number of total mutations, single nucleotide polymorphisms, indels, non-silent mutations and the tumor size. Median and interquartile range are represented in figure. (B) Scatter diagram showing the linear correlation between tumor size and total number of mutations, as well as between tumor size and number of non-silent coding mutations. Blue dots: panNETs ≤2 cm; orange squares: panNETs >2 cm.

**Figure S3 – Mutation frequency in panNETs ≤2 cm and >2 cm.** Frequency of gene mutations in panNETs ≤2 cm and >2 cm from the Scarpa’s cohort [12]. Mutations of *DAXX* were reported in 1/14 panNETs ≤2 cm and 21/71 panNETs >2 cm (*p*= 0.1). Overall, a mutational frequency of 41.2%, 25.9% and 3.5% was reported for *MEN1*, *DAXX* and *TSC2* respectively.


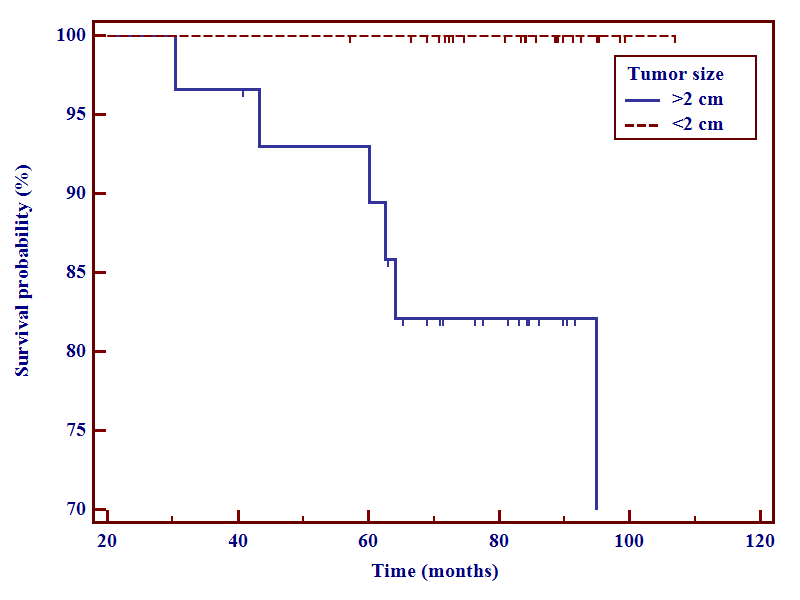

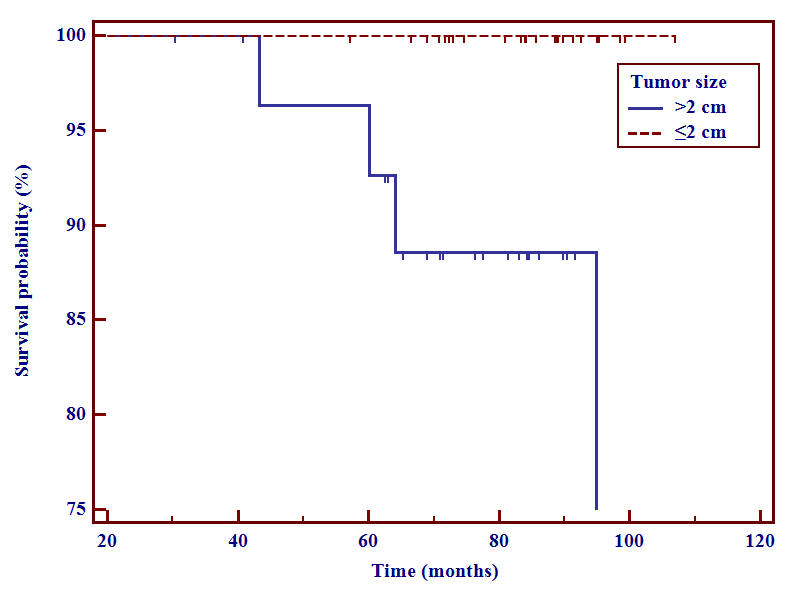


**A**

**B**

***p*=0.01**

***p*=0.05**


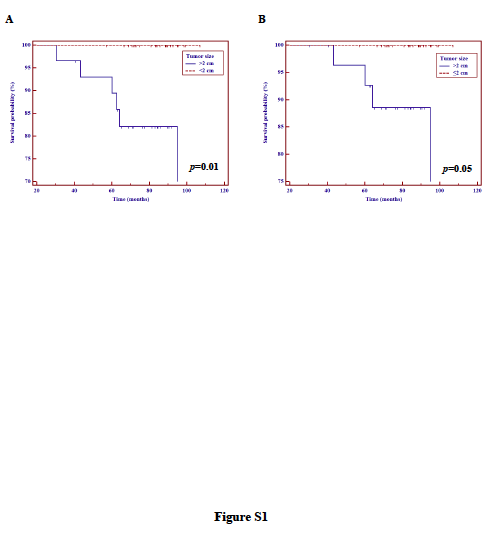


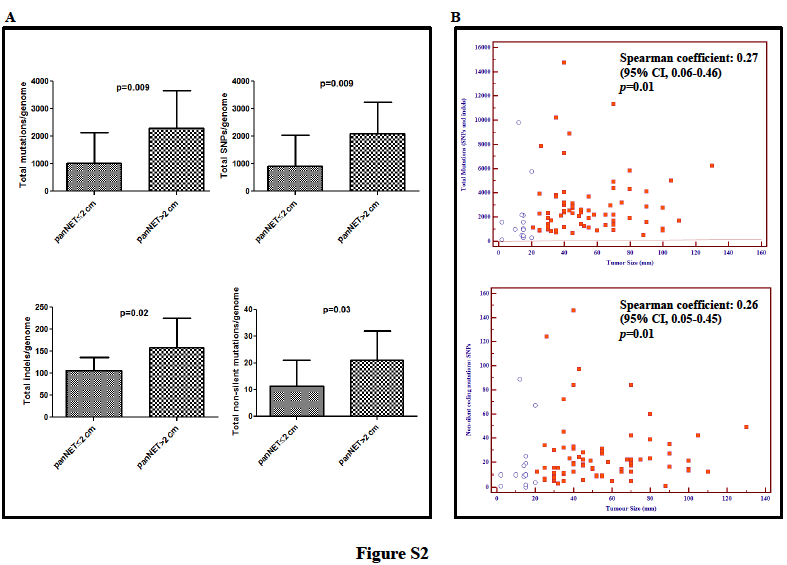


10

0

20

30

40

**Frequency of somatic mutations (%)**

**MEN1**

**DAXX**

**TSC2**

**BCOR**

**ARID1A**

**ATRX**

**CDC42BPB**

**DST**

**PRRC2A**

**ZNF292**

**SETD2**

**TP53**

**DEPDC5**

**KLF7**

**PIK3CA**

**PTEN**

**≤2 cm**

**>2 cm**


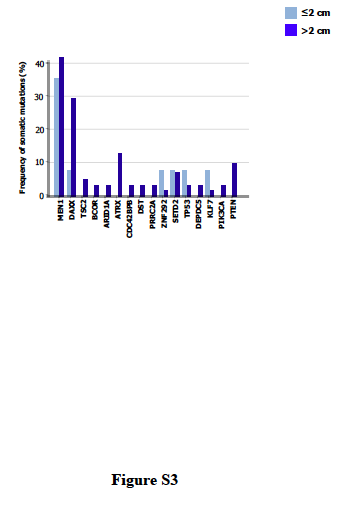

Supplement: Supplementary file 1 — Supplementary information [file 41598_2019_55156_MOESM1_ESM.docx]
